# Mentoring in the Time of COVID‐19: An Analysis of Online Focus Groups with Mentors to Youth

**DOI:** 10.1002/ajcp.12543

**Published:** 2021-07-28

**Authors:** Michelle R. Kaufman, Kate Wright, Jeannette Simon, Giselle Edwards, Johannes Thrul, David L. DuBois

**Affiliations:** ^1^ Department of Health, Behavior & Society Johns Hopkins Bloomberg School of Public Health Baltimore MD USA; ^2^ Ananizach, LLC Gwynn Oak MD USA; ^3^ Department of Mental Health Johns Hopkins Bloomberg School of Public Health Baltimore MD USA; ^4^ Centre for Alcohol Policy Research La Trobe University Melbourne Vic Australia; ^5^ Department of Psychology School of Public Health and Institute for Health Research and Policy University of Illinois Chicago Chicago IL USA

**Keywords:** COVID‐19, E‐Mentoring, Mentoring, Online focus group, Youth

## Abstract

This study explored the experiences of mentors to youth during the early months of the COVID‐19 pandemic. The study aims were to examine (1) the role of the pandemic on mentor–mentee interactions and relationships and (2) the ways in which mentors could be supported during the health crisis to better meet youth needs. Six online focus groups were conducted with 39 mentors. Mentor participants included 26 females and 11 males (two did not disclose gender), and 51% identified as white. Any mentor currently in a mentoring relationship, regardless of type, was eligible. Using Facebook groups, moderators posted questions and prompts, and mentor participants responded using textual comments. The text from each group was recorded, extracted, and coded and analyzed using thematic analysis. As mentors transitioned to a primarily online format, text and video chat became the most common communication methods. Mentees’ access to technology and privacy were the biggest challenges faced. Mentor concerns for their mentees varied, including mental health, school, family finances, and access to instrumental support and food. Mentor help involved routinely connecting with mentees and providing academic support. Mentors requested ideas and resources for connecting with mentees and an online mentor support group. During the early weeks of the pandemic, mentors continued to engage with mentees, offering valuable support during a confusing and scary time. Mentoring programs can broaden their approach, intentionally integrating online connecting in an effort to provide safe, appropriate, and continued support to both mentors and mentees.

## Highlights

- Mentors connected with mentees early in the pandemic, portraying dedication to their relationships.
- Mentoring can adapt to virtual operations, despite difficulties in transitioning to a new format.
- Virtually connecting with mentees can be difficult for mentors without prior, organized planning.
- Mentors need support during the pandemic through meaningful resources or virtual support groups.

## Introduction

Youth mentoring involves nonparental adults (or older peers) providing young persons with support in a one‐to‐one or small group relational context with the aim of promoting positive development and well‐being (Dubois & Karcher, 2013; Raposa et al., 2019). Most conceptualizations of mentoring for youth focus on relationships occurring outside of professional helping contexts such as counseling or psychotherapy (Dubois & Karcher, 2013). Rhodes’ (2005) influential model of youth mentoring posits that mentors are able to facilitate positive developmental outcomes via processes that support the youth’s socio‐emotional, cognitive, and identity development (e.g., role modeling, scaffolding for skill acquisition, expanding conceptions of possible educational and occupational goals). These developmental gains, in turn, are expected to strengthen the young person’s overall web of support via enhanced relationships with parents, other adults (e.g., teachers), and peers, thus further increasing the potential for mentoring relationships to contribute to positive outcomes. In line with these possibilities, research indicates that participation in formal mentoring programs can positively impact youth in a number of ways, including protection against aggression, depressive symptoms, delinquent behaviors, victimization, and substance use, as well as advancing cognitive and identity development (Brezina et al., 2016; DeWit et al., 2016; Raposa et al., 2019).

Young people can learn to manage their emotions and use effective coping strategies by observing and experiencing appropriate communication and pro‐social behavior modeled by adult mentors. Additionally, the benefits of a close and consistent attachment to an adult can reduce feelings of social isolation and loneliness (Keller et al., 2020) and create views of self and others that facilitate positive interpersonal relationships (DeWit et al., 2016). Mentoring relationships often include activities and interactions that provide opportunity for intellectual challenges and positive learning experiences, which can promote self‐esteem (DeWit et al., 2016). Observed benefits are variable, however, both within and across programs, with research pointing to mentor training and ongoing supervision, regular mentor–mentee meetings, and incorporation of opportunities for mentors to serve in informal teaching and advocacy roles as practices that may promote better outcomes (DuBois et al., 2002; DuBois et al., 2011; Garringer et al., 2015; Tolan et al., 2014).

A wide variety of formal mentoring programs exist. Some programs operate within a school setting, providing support throughout the school day and/or within the school building. Community programs reach youth outside of a school setting and offer a safe space away from both home and school for the mentor and mentee to develop their relationship (Garringer et al., 2017). Some programs operate primarily through group interaction, involving one or more mentor engaging with more than one mentee at a time (Kuperminc & Deutsch, 2021), whereas one‐on‐one mentoring models match one specific mentor with one specific mentee, creating an intentionally individual relationship.

### The COVID‐19 pandemic

In March 2020, during the beginning of the country’s COVID‐19 pandemic, much of the United States declared an emergency stay‐at‐home order in which social distancing was required except for those in the same household. Many employees lost their jobs while others worked from home alongside their children who finished the school year remotely (Galea & Abdalla, 2020; Hale et al., 2020). For public health purposes, the situation required social isolation, creating a potentially harmful environment for some youth already at risk for mental health concerns. Social isolation can lead to low self‐esteem, depressive symptoms, abuse, and suicidal ideation (Hall‐Lande et al., 2006; Hazler & Denham, 2002), particularly for adolescents who rely heavily on peer interactions—all of which can be positively impacted through mentoring (DeWit et al., 2016; King et al., 2002).

Youth are in a developmentally sensitive stage of their lives, where youth‐serving institutions are critical for peer engagement, development of self‐identity, and acquiring social and emotional skills (Curran & Wexler, 2017). School closures due to the pandemic are a cause for concern because they specifically disrupt routine activities and access to important services (such as food and mental health care) provided by these institutions. Without peer support, a stable routine, and school services, youth lose their anchor for coping with issues such as anxiety and depression (Courtney et al., 2020; Golberstein et al., 2020; Lee, 2020).

Recent reports show an increase in psychological distress and mental health conditions since the pandemic began, including for youth. Risk factors for increased psychological distress include the following: a history of anxiety or depression (Holingue et al., 2020b; Riehm et al., 2020); consumption of alcohol or cannabis (Holingue et al., 2020b); more time spent on social media focusing on COVID‐19 (Riehm et al., 2020); and living in a household that has experienced reduced income or work hours (Holingue et al., 2020a). Specific populations more likely to report an increase in mental health conditions include younger adults (ages 18–24), racial/ethnic minorities, essential workers, and unpaid adult caregivers (Czeisler et al., 2020). Young people are particularly at risk for negative mental health outcomes during the pandemic (Guessoum et al., 2020; Oosterhoff et al., 2020).

Mentoring has demonstrated the potential to benefit youth in times of crisis. For instance, therapeutic mentoring has shown promise for reducing the impact of trauma for youth in foster care (Johnson & Pryce, 2013). Other research similarly points to the potential for mentoring to benefit youth with parents who are incarcerated (Jarjoura, 2016), youth who are refugees or recent immigrants (Oberoi, 2016), youth whose parents are connected with the military (Basualdo‐Delmonico & Herrera, 2014; Spencer et al., 2020), youth transitioning from foster care (Collins et al., 2009; Taussig & Weiler, 2017), and for those reentering their schools and communities after juvenile confinement (Eddy & Schumer, 2016). Thus, although research does not exist on the effectiveness of mentoring during a global pandemic, there is indirect evidence to suggest that the continuity of a mentoring relationship during such a time of crisis may be highly beneficial.

### The State of E‐Mentoring Prior to the Pandemic

E‐mentoring, or digital or virtual youth mentoring, is a fairly nascent field but has grown in popularity due to the proliferation of digital media and the widespread usage by adolescents (Rideout & Robb, 2019; Shpigelman, 2014). While some tech‐based mentoring programs have been using digital technology with youth mentees for years, e‐mentoring was mostly limited to special populations prior to the pandemic, such as youth with disabilities, and to programs oriented toward career development in areas such as STEM (Kaufman, 2017). Small‐scale demonstration and pilot studies of these types of programs have reported some preliminary evidence of effectiveness (Kaufman et al., 2020; Kaufman, 2017). More recently, formative research also has highlighted youth with stigmatized identities (such as sexual and gender minority youth) as being receptive to digital platforms as a way to connect with mentors discreetly without needing to meet in person (Kaufman et al., 2020). In summary, although research on e‐mentoring for youth is in a nascent stage, it shows promise for offering a range of possible benefits (Kaufman et al., 2020).

Given the increased vulnerabilities of youth and the limited knowledge on how e‐mentoring is used broadly within the mentoring field, the goal of the present study was to determine how mentoring pairs adapted to the new pandemic reality. Through online focus group (OFG) discussions with mentors about their experiences, perceptions, and needs, this study intended to answer the following research questions: (1) what was the impact of the pandemic on mentor–mentee interactions and relationships, and (2) how could mentors be supported during the health crisis to better meet youth needs?

## Method

This study was approved by the Johns Hopkins Bloomberg School of Public Health Institutional Review Board.

### Recruitment

Mentors were recruited from a variety of locations and mentoring programs that served multiple youth subpopulations. We did not specify a particular mentoring model or setting because we wanted the results to be as generalizable as possible given the qualitative nature of the study. Using a digital flyer, recruitment occurred through outreach to mentoring organizations by both the research team and MENTOR: The National Mentoring Partnership, emails to mentoring research and practice listservs, outreach via public Facebook groups focused on mentoring, and other social media outlets (Twitter, LinkedIn). Mentoring organizations also distributed study information to their mentors.

Prospective participants needed to meet the following criteria: age 18+; living in the United States; currently acting as a mentor to a young person (age 18 or younger) for at least three months; affiliated with a formal mentoring program; have a Facebook account (or willing to create one); and access to a mobile device with Internet connectivity.

The flyer directed interested participants to a Qualtrics survey to determine eligibility. Eligible participants were directed to an online consent form. Following consent, each participant was requested to register for a scheduled focus group. Each participant provided a Facebook handle and email address for communication.

Participants were invited to join a Facebook private group for the date and time they chose. Only group members and discussion facilitators had access to information in the group. If any participants did not take part on the chosen date, their profiles were removed from the group so they could not access the data, and they were invited to join a future session. One participant from each OFG was chosen randomly to receive a $50 gift card.

### Participants

**Fig. 1:**
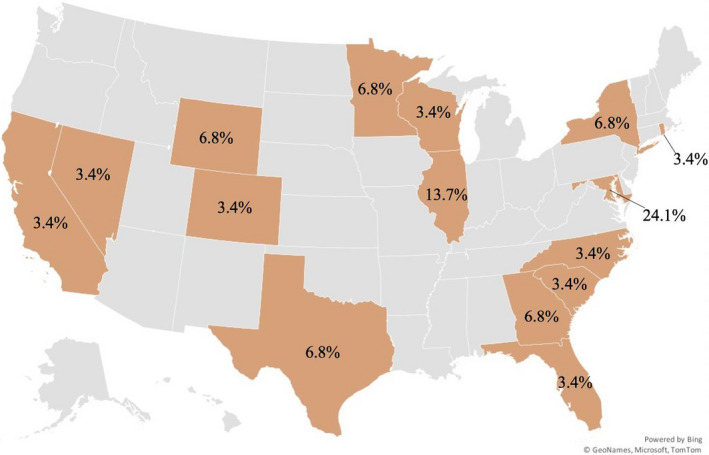
States represented by mentor focus group participants. Note: This map only includes data on 75% of participants, as 25% did not indicate their location. In addition, Maryland disproportionately represents a quarter of the geographic data due to the research team’s location, visibility in the state, and their personal connections with local mentoring organizations

A total of 39 mentors participated. OFGs included male (*n* = 11) and female (*n* = 26) participants (and two participants who did not state their gender identity) from across the country (see Fig. 1) who identified as white (51%), Black (23%), Latinx (10%), and Asian American (7.5%). One participant identified as both white and Latinx. Five out of six OFGs had much stronger female representation, with one male participant in three OFGs and no male voice in two OFGs. The sixth OFG had more male participants, with eight males, five females, and one participant with undisclosed gender identity. This group also had the highest number of participants overall, representing 36% of total participation. For details on demographics of mentors, see Table 1.

**Table 1:** Mentor gender and racial/ethnic characteristics

	Males	Females	Undisclosed	Black	White	Asian	Latinx	Undisclosed
OFG1	1	2		3				
OFG2	1	4	1	1	4			1
OFG3	1	8		2	4	1	2	
OFG4		3		1	2		1	
OFG5	8	5	1	1	9	2		2
OFG6		4		1	1			2
Total	11	26	2	9	20	3	3	5

OFG, online focus group; participants could check more than one racial/ethnic category.

Mentors came from school and community‐based programs as well as one‐on‐one and group mentoring models. Their mentees ranged in age from 4–25 years (some mentors had younger mentees that allowed them to meet study eligibility criteria but also mentored older youth). Mentee characteristics were requested but not required as part of the eligibility form, and many mentors chose to decline providing demographic details for their mentees to protect the youth’s anonymity. We received very few details on race or family socioeconomic status, in particular. Of the available data, mentors discussed 19 female and 7 male mentees, including one 6‐year‐old, one 9‐year old, 17 10‐ to 14‐year olds, and 20 mentees ages 15 and older. Many mentors stated they worked with multiple mentees and provided an age range, such as 11–19 or 4–18; therefore, defining exact numbers was difficult.

### Procedures

Qualitative data were collected in April 2020 (early in the U.S. COVID‐19 pandemic) using OFGs conducted in Facebook private groups. Participants used their Facebook profiles to take part in the discussion; therefore, all members of each private group could identify each other and view any public content associated with their profiles.

All data were text conversations (rather than video interactions). Procedures for the OFGs were based on the methodology utilized by Thrul et al. (2017) and Ramo et al. (2019) and used a phenomenological approach (Bernard, 2006) with the expectation of gaining an understanding of the participating mentors’ experiences. Six OFGs were conducted over the course of three weeks. Each OFG lasted approximately 60 minutes and provided opportunities for participants to comment on direct questions posted by the facilitators as well as to interact with other posts.

During the OFGs, group guidelines were posted first. Study team members (described below) then led a discussion focusing on whether and how participating mentors were continuing their relationships with mentees during the pandemic. Mentors discussed any current mentoring relationship that was applicable without the requirement of choosing one in particular, thus allowing for open conversation about varied experiences from each participant. The discussion guide (see Table 2) was designed by the authors and asked about concerns that may arise for mentors of youth during the pandemic and/or quarantine. These discussions delved into frequency and type of communication since social distancing policies were instituted. Mentors also provided information about any changes in their approach to mentoring as well as whether and how health or safety topics were being incorporated into their relationships and/or into programming efforts.

**Table 2:** Online focus group guide

Focus Group Questions	Probing Questions
1. Have you been in touch with your mentee since the start of social distancing?	How often do you communicate? How do you communicate? Has your mentee reached out to you during this time? Do you communicate more or less often with your mentee during this time than other times?
2. What questions have your mentees asked you about the Coronavirus or issues related to it?	What is your mentee’s general feeling about the Coronavirus and social distancing?
3. How have you been supporting your mentee related to the Coronavirus pandemic?	Have you provided information? If so, what type? Have you offered other types of support? If so, what kind? Are you encouraging your mentee to engage in social distancing? What are you suggesting they do?
4. Is there anything your mentee is especially concerned or worried about?	
5. Is there anything you are worried about for your mentee or their family?	Physical health concerns? Mental health concerns? Financial implications due to widespread closures? Food insecurity due to school closures? Impact on academics due to school closures? Challenges in their home environment?
6. What would help to make you a better mentor during this challenging time?	
7. What information would you like to have to pass on to your mentee during this time?	Are you interested in learning about resources related to Coronavirus or social distancing? Are you interested in learning about how to use technology better to maintain the relationship with your mentee?
8. Is there anything else you want to share regarding your mentoring experience during the pandemic?	

The same three moderators administered each OFG. Moderator 1 (MK) is an academic researcher with expertise in youth mentoring and experience serving as a mentor in Big Brothers/Big Sisters. Moderator 2 (JS) is a mentoring program expert with over 25 years of experience leading and providing technical assistance to mentoring programs across the country. Moderator 3 (KW) has expertise in qualitative data collection with vulnerable populations.

Moderator 1 posted the main focus group questions; Moderators 2 and 3 asked follow‐up questions and reacted to participants’ comments through “likes” and replies. Each question was posted approximately five minutes apart to allow participants to respond and interact while at the same time maintaining engagement if they responded immediately. Participants had the opportunity to continue responding to questions and comments after the OFG ended for a period of 24 hours. Few comments were made during this time, and most occurred within the first 15 minutes following the session.

### Analysis

Written comments were copied into a Word document and uploaded to ATLAS.ti for coding and analysis. All extracted Facebook content was anonymized by replacing participant names with IDs and deleting profile pictures. A codebook was created based on the discussion guide and study objectives prior to coding. Two study team members (KW and GE) coded the data independently, initially using a deductive approach with pre‐determined codes and then using an inductive approach as the coders gained more familiarity with the data. A few codes were added, specifically relating to mentee concerns, such as social distancing and finances. Team members made constant comparisons throughout the coding process in order to ensure consistency of meaning and to denote any changes in the interpretation of codes (Gibbs, 2007).

An initial reliability check was performed with an 80% result. One study team member (KW) initially reviewed the discrepancies and made changes to her own codes, where appropriate, to match the other team member’s (GE) codes. Further discrepancies were then discussed between the two coders until a consensus was reached, specifically noting any codes with consistent disagreement throughout the transcripts. For example, the two coders did not always agree on the codes for concern for mentees and support for mentees. This discrepancy showed a potential overlap between these two codes. In addition, KW coded resources more often than GE. Reviewing the data again, 92% agreement was achieved. Resource codes by KW were left in, and KW’s codes were used for other remaining code discrepancies as well. This overall approach to coding helped to define the meaning of the codes and to prevent bias and ensure consistency and reliability (Gibbs, 2007).

Thematic content analysis (Anderson, 2007; Erlingsson & Brysiewicz, 2013) was performed by the same team members (KW and GE) by reviewing the codebook and identifying common themes in the coded data. Relevant sets of codes were then grouped together to establish prominent meaning, thus creating themes. The data were read and reread multiple times, with checks made on previous groupings to confirm relevancy and consistency. Thematic frequency, connections, and patterns were used to further categorize the data and discern relationships. Team members noted themes that occurred more often or that regularly appeared alongside other themes within a smaller subset of data, implying significance of certain themes and connection among sets of themes. Reading through the data set multiple times provided the opportunity to obtain a solid understanding of meaning, resulting in a picture of fluidity and interconnectedness among themes while also establishing separate categories of meaning. For example, two themes—mentor concerns and mentor support for their mentees—directly impacted each other but remained distinctly significant.

## Results

Four themes arose throughout the OFGs: communication patterns during the pandemic, mentor concerns, types of support mentors provided to their mentees, and mentor needs. The first three themes showed clear signs of interconnectedness and answered the first research question about the impact of the pandemic on mentor–mentee relationships—communication patterns portrayed different means of support, mentor concerns related to efforts of communication, and mentors discussed attempts to relieve some of the concerns they felt for their mentees through specific methods of support. The final theme answered the second research question regarding what mentors required during the health crisis to better meet youth needs. This theme showed some connection to the other three, specifically in mentors’ requests for new ideas for ways to interact with mentees but stood more firmly on its own as their personal needs as mentors did not always relate directly back to their experiences with mentees during this time. Below we unpack each theme in detail.

### Communication Patterns During the Pandemic

#### Frequency and Mode

**Fig. 2:**
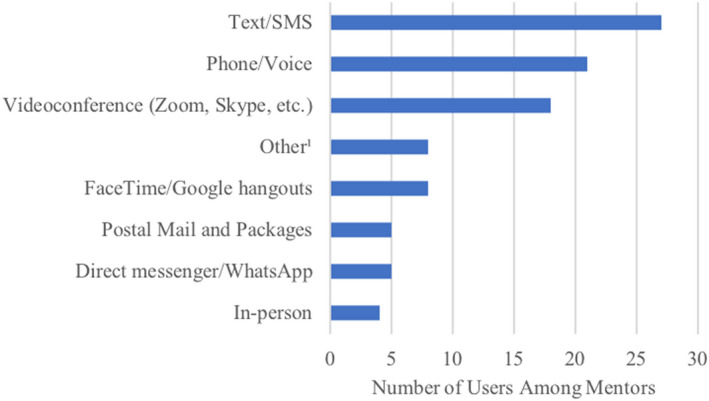
Mentor–Mentee communication methods during the COVID‐19 pandemic.^1^The “other” category includes alternative forms of technology mentioned. These include the use of apps such as Marco Polo, House Party, and Heads Up. Additionally, it incorporates online games such as Pictionary, 20 Questions, and Would You Rather and online joint activities such as Netflix Party, Dolly Parton’s bedtime stories, online drawing lessons or book clubs, video games, dancing, and cooking. The games and joint activities were conducted through video or other platforms

Mentors shared that they continued to connect with their mentees at a similar frequency as before the pandemic, although communication methods changed due to stay‐at‐home orders and social distancing guidelines (see Fig. 2). The incorporation of digital technology, apps, and creativity in meeting via online platforms was crucial.

We’ve used the app called House Party—it’s like FaceTime but you can play games at the same time (like trivia, Apples to Apples, Heads Up, and Pictionary). (female, California)

Some participants delivered supplies to their mentees or saw them in person with proper precautions. These interactions were far less common than through online means.

#### Communication Challenges

Mentors explained difficulties they faced with communicating during this time. Some mentors shared that their mentees did not have access to a phone or computer, so the more common online communication methods did not apply to them, resulting in less frequent communication.

Although it’s hard to stay in touch, I try my best [now] that I have signed up to be in her life. (female, Florida)

Some mentors found it difficult to connect with their mentees at all in these cases and would sometimes go by their homes simply to “*lay eyes on everyone*” (female, Maryland) and make sure they were okay. Some families did not have Internet access or shared one phone, and mentors had to plan conversations with the mentee’s parent, creating an extra scheduling barrier.

Unfortunately the digital divide is deeply impacting the most vulnerable youth in our community. If you don’t have technology or communication resources and can’t leave home, you can’t learn, you can’t get help or support, and you can’t be mentored. (female, Minnesota)

Multiple participants also discussed the struggle to communicate openly and easily with their mentees through digital means compared to previous in‐person meetings. Mentors felt that some mentees, particularly some ages, felt uncomfortable talking over the phone or on video.

Others found it difficult to connect with their mentee because the mentee’s living situation did not allow for privacy; therefore, in‐depth conversations were less likely to occur. Sometimes, this lack of privacy, alongside changing school and work expectations, made it hard to schedule a time to connect.

It’s harder for children to be open with you via phone than if you were together. The concern of someone hearing you will prohibit people from being completely open. I feel strongly that she won’t share her mental state with me unless she is in complete privacy. (female, Florida)

### Mentor Concerns

Mentors cited a long list of concerns for their mentees.

We have a number of families that are really struggling financially. Very food insecure, don’t have the technology they need for distance learning, and are dealing with their very immediate challenges and crises. (female, Minnesota)

#### Mental Health

Mental health concerns focused on the isolation the pandemic created and the close quarters families often lived in, inherently constraining privacy and potentially limiting youth’s freedom of expression. The greatest concern was expressed for mentees with depression or previous suicidal ideation. Some mentors felt unease regarding adult substance use or potential physical or mental abuse within the home and the lack of access to support or help, if necessary.

I have mentees where it’s literally brain damage to sit all day in a home with a parent that verbally and emotionally abuses them. (gender unknown, South Carolina)

#### Food Insecurity

Mentors expressed concern about mentees and their families having insufficient food when schools and other community organizations were temporarily closed.

I worry that there may be other consequences on the other side of this, not enough income can lead to bills not being paid or food not being in the pantry. And I just hope they have the resources to not put them in an even more vulnerable place because they are already vulnerable. (female, Maryland)

Some mentors talked about dropping off groceries to mentees’ homes to fill gaps left by mass closures.

#### Physical Health

In addition to worry about decreased access to healthy foods, especially in homes where meals were often provided by schools, there was concern about a lack of exercise or outdoor activity.

I’m worried about my mentee not getting enough exercise—I know she watches a lot of TV/plays a lot of video games, and with her inability to go outside I’m worried this is even worse. (female, Illinois)

Other physical concerns were related to COVID‐19, such as potential virus exposure through working parents or teenage siblings who may not carefully observe prevention measures.

Their mom works in a place that hasn’t been following the rules very well. We had to supply her masks. I’m terrified she’ll bring it home to them. And their apartment complex is overcrowded. (female, Maryland)

### Mentors as Support

Participants in the OFGs went above and beyond what was expected by the mentoring programs while also striving to set appropriate boundaries. The pandemic created different needs for many mentees, requiring mentors to shift their focus in order to provide support most effectively. Keeping in touch allowed mentors to better understand what each mentee needed and to adjust accordingly. The OFG with a stronger male voice interacted less with the discussion surrounding support for mentees, specifically in regard to providing helpful information, such as sharing resources. Interestingly, this OFG also provided the majority of comments in relation to mentors needing more information and resources.

#### Instrumental Support

Mentors discussed acting in ways that provided solutions to specific concerns or struggles. Outside of online connections, some mentors delivered items to their mentees’ homes—groceries, masks, laptops, school packets, arts and crafts, games, and other fun activities. Many provided school support through tutoring, correcting schoolwork, or helping to navigate online learning and to keep a schedule. Some offered assistance to the entire family by helping with job applications, health insurance concerns, or receiving stimulus checks. In addition, they often explored and shared community resources for where to receive free services, such as meals, laundry, and Wi‐Fi.

When my mentee tells me his family needs something, I connect them with the Latin American Association who have some programs to support the Latino community. (male, Georgia)

#### Emotional Support

Mentors and mentees discussed various topics during the health crisis, and mentors described a desire to support their mentees through whatever conversations with which they felt comfortable. Some talks centered around COVID‐19—discussions about safe behaviors, social distancing, masks, and the stay‐at‐home order; the reality of stress during the pandemic; and what life might look like on the other side of COVID. But sometimes mentees wanted to talk about normal things, with no mention of the pandemic at all. Mentors endeavored to create a safe environment for their mentees and did not force any unwanted conversations.

Our time together can be a break from life and feel back to normal since we are doing similar things to when we were in person. (female, Maryland)

Even with the changing circumstances and added effort, some mentors expressed a wish to do more. Many participants agreed or voiced a similar idea, struggling with maintaining proper boundaries:

Everything is more difficult. Even little things you take for granted are plagued by questions of concern for supply, mental health, or even feeling adequate to figure things out. I think we have to just be confident that we can do this no matter what is thrown our way… The relationship is important and I think you have to quit trying to be perfect and just be what you can be. Your mentee values you more than you realize. (female, Wyoming)

#### Creative Companionship

Some mentors described doing physical activities together over video, such as online exercising or dance parties. Other online activities focused on skill‐building, from sewing homemade masks to life skills for coping with difficult times to dealing with police encounters during a time of social restrictions and social unrest.

I also try to keep his mind off the stress of the pandemic by sending him videos and links about our shared favorite pastime: basketball. (male, Illinois)

### Mentor Needs

The OFGs brought out a variety of ideas that mentors felt would help them fulfill their role during this time. The two most frequent requests for support were discovering new ways to connect with their mentees during a pandemic and being part of a support group for mentors. The OFG with heavier male participation mentioned needing ideas for connecting with mentees at a higher rate than the other OFGs.

#### Ideas for Safe Activities

After six weeks of mentoring online, mentors were beginning to search for non‐tech activities to engage in with their mentees. They were particularly interested in safe, outdoor activities. In addition, mentors felt that exploring new ideas to connect online with new mentees was vital.

I’m thankful for technology during this time, but I also think it puts a spotlight on new forms of mentoring that need to be developed. 1‐on‐1 mentoring…isn’t the norm anymore. The more challenges are introduced, the more ways we need to evolve. (male, North Carolina)

#### Mentor Support Groups

Across the board, participants agreed that an online support group for mentors would be incredibly helpful. Sharing ideas, discussing experiences, and connecting with other mentors about their own stress and anxiety would provide a much‐needed outlet and resource during such unprecedented times. Some participants stated the OFGs felt helpful in this way:

Seeing everyone’s responses here has reminded me that we’re not alone in our work/struggles in being a mentor. (female, Illinois)

Others expressed that continuous collaboration with others could provide encouragement and strength, contributing to their own health as well as their effectiveness as mentors.

A support group where we can join together to share thoughts and experiences with one another. What if the group included mastermind sessions where, as a team, we examined our experiences to identify possible solutions and to be reminded that we are not alone? (female, Maryland)

#### Additional Resources

Other requests revolved around resources: reliable and age‐appropriate COVID‐19 information to share with their mentees; an emergency preparedness guide for young people; COVID‐19‐adapted mindfulness resources for youth; and positive examples for overcoming crises that could be shared with mentees. Other resource requests included information on available services for families; educational resources outside of school; and self‐care for mentors. In line with their focus on education, mentors also thought it would be helpful to have direct communication with teachers to better assist with schoolwork.

## Discussion

Mentors in this study continued to connect with their mentees despite the difficulties created by the pandemic. They seemed dedicated to their relationships and determined to adjust their mentoring practices to suit the needs of the pandemic and each mentee as an individual. Their extra effort showed commitment, intentionality, and depth—and that mentoring programs can adapt to digital operations. The OFGs proved how difficult it can be to connect with mentees. A thoughtful and thorough approach to creating a safe and comfortable space can result in meaningful connections, but understanding and implementing best practices for connecting online is necessary in order to avoid an early dissolution of relationships (Garringer et al., 2019; Kaufman, 2017; Miller & Griffiths, 2005; Shpigelman, 2014).

Mentoring has proven beneficial for youth who feel isolated (Keller et al., 2020), and stable, long‐term relationships stand to have a very powerful effect for mentees (DeWit et al., 2016). Many mentors in this study clearly understood that consistency within their mentor–mentee relationships was important, and their concerns for and attempts to address physical and mental health needs portrayed how mentors can have a positive impact on the lives of their mentees. This may have been especially important during the pandemic given many youth had reduced opportunities for social‐emotional interaction due to necessary school closures and social distancing. Despite this interruption during a critical youth developmental stage, mentors had the opportunity to fill the gap, and those participating in this study stepped up in many instances.

More broadly, it remains to be seen whether youth who were able to maintain a relationship with a mentor during the pandemic fared better in their emotional well‐being compared to those youth without the stability of such supportive relationships. Youth without both peer support and healthy ways to cope with mental distress, such as the guidance of a supportive adult, are more likely to experience anxiety and depression (Courtney et al., 2020; Golberstein et al., 2020; Lee, 2020). As a result, the mental health of youth has been of growing concern as a result of the pandemic (Rousseau & Miconi, 2020). However, mentorship presented an opportunity to mitigate the stress associated with a global crisis. While the study of the impact of youth mentorship during a global health crisis is new, there is evidence showing that such relationships have been helpful in similar contexts, such as the prevention of HIV (Kaufman et al., 2020), and in crisis situations, such as the recovery from traumatic events (Johnson & Pryce, 2013).

Mentors need growing support as they, too, are living through a pandemic. A support group would help mentors in the moment, but it would also create a larger community, connected not only through an organization but through the practice of and passion for mentoring itself. Such a community could promote new ideas while contributing to mentors’ confidence, continued interest, and overall mental health, thereby encouraging mentoring relationships overall (Garvey & Alred, 2000; Marshall et al., 2015; Stukas & Tanti, 2005; Thornton, 2014).

As the pandemic subsides but the threat of similar crises in the future remains, it is important to ensure mentors have the skills and tools necessary to provide appropriate assistance and referrals for mentee needs in such situations. Adaptive programming will be crucial for mentors as needs evolve and new difficulties arise. Mentoring programs as well as individual mentors must take the lessons of COVID‐19 forward, finding ways to be flexible during unexpected challenges.

### Strengths, Limitations, and Future Directions

This study contributes to the current needs of the world in the midst of the COVID‐19 pandemic and can provide guidance for mentors as well as programs and researchers, identifying ways to use technology to support mentees and mentor–mentee relationships. The results of this study can also be used to prepare for future unexpected difficulties that require digital communication. Because data for this study were collected during the pandemic, the opinions and suggestions of the mentors were fresh and current.

The results of this study should be considered in light of their limitations. Due to the time‐sensitivity of the topic, youth were not included in this research; therefore, we only heard mentors’ perspectives. In addition, mentor participants were self‐selected and so may have been particularly motivated mentors. We do not know the status of mentoring relationships where the mentor had their own challenges during the pandemic’s early stages that might have interfered with their ability to mentor, let alone participate in a research study. These results are not generalizable to those beyond the mentors who participated. Participants also represented a broad range of mentoring programs, as opposed to one specific type, making the results difficult to translate into strategy for any one type of program.

Communication in a text format, as opposed to verbal communication, may result in a less comprehensive data set. An OFG allows for participants to take part at their own pace, which may lead to participants interacting with the conversation at different rates, reducing the amount of real personal interaction that may be more likely to occur in a verbal format. Similarly, though OFG moderators were responding to comments with follow‐up questions, this format does not provide as much opportunity to probe participants as a verbal focus group may allow. As a result, it is possible that the data are less informative than with an in person or online, video‐style focus group where participants and moderators are “face‐to‐face” and interacting verbally with one another. In addition, the use of Facebook may create data privacy concerns, even with the use of private groups. Lastly, the initial OFGs took place very early on in the pandemic, and a follow‐up OFG would have been helpful for understanding longer‐term adjustments and needs.

Despite these limitations, the use of OFGs was successful in helping us to quickly understand the challenges that were occurring in mentoring relationships and what further resources and support were needed to ensure these relationships endured this public health crisis. Previous research has shown e‐mentoring success with a variety of subpopulations (Cantrell et al., 2010; Cassiani et al., 2020; Gregg et al., 2016; Kaufman, 2017; Li et al., 2010; Merrill et al., 2016; Merrill et al., 2015; Stoeger et al., 2013), although e‐mentoring programs are rare with the general population overall, accounting for only three percent of mentoring interactions (Garringer et al., 2017). Future research may explore what elements of a digital relationship make for strong e‐mentoring. For instance, researchers could assess textual chats between mentors and mentees or code video calls to determine the qualities of a meaningful digital relationship (or identify what is counterproductive). Investigations should also assess whether e‐mentoring differs in its effectiveness compared to in‐person mentoring (or no mentoring at all) for youth more broadly. Finally, it would be useful to know if there are differences in the effectiveness of e‐mentoring across youth age groups. For instance, younger youth may be more difficult to engage via digital means, whereas teenagers may be more comfortable engaging in this way as compared to in person because they are used to communicating via digital technology with their peers.

### Implications

Ensuring consistent connection between mentors and mentees is vital, regardless of circumstances. Programs can develop emergency plans in an effort to prepare for unexpected events with the intention of ensuring continuous support for mentees. Informed, comprehensive digital mentoring platforms can provide support during potential future shutdowns while also offering a new way of mentoring on a regular basis. In addition, it would be beneficial for the entire mentoring field if programs had a tool to assess whether they have sufficient capabilities for transitioning to e‐mentoring. Many programs were blindsided by the need for a sudden transition. More purposeful planning for using e‐mentoring would strengthen these programs in the future.

During this pandemic, mentors can benefit from a list of ideas for safe and appropriate ways of connecting with mentees, both in person and digitally, as the current situation allows. Such a resource can encourage continuous connection as well as conversations around safe and healthy choices regarding changing COVID restrictions. Finally, cultivating and encouraging a way for mentors to connect with each other can have a lasting positive impact on the experiences and longevity of mentors. A place for mentors to connect was suggested in this study in response to the COVID‐19 pandemic, but mentors can continue to learn from and support others beyond this time of crisis.

The pandemic highlighted the potential for new technologies to stretch the reach of mentoring overall, expanding the number of youth who can access a mentor (Garringer et al., 2015; Kaufman, 2017). Mentors in this study noted that, once the technology was in place, they were able to have fun and meaningful interactions with their mentees. More specific guidance is required for mentors to choose the most appropriate activities, duration, and frequency of e‐mentoring interactions, depending on the needs and developmental stages of their mentees. That being said, mentors and their programs adapted quickly and effectively, opening the door for mentoring programs to offer e‐mentoring beyond what the pandemic necessitated. Digital forms of mentoring have the potential to reach more youth and can provide more opportunities for mentees to connect with mentors who have certain characteristics and/or a shared lived experience and who may not be living in the mentee’s immediate community. For example, youth who have a rare health condition may require specific characteristics in a mentor, or a female mentee might expressly like a female mentor in engineering or computer science (Kaufman et al., 2020). E‐mentoring also has the potential to expand the number of mentors, as it provides flexibility when transportation time and costs are removed or when mentor–mentee interactions can occur asynchronously (Kaufman, 2017; Kaufman et al., 2020).

### Conclusion

During the early weeks of the COVID‐19 pandemic, mentors intentionally remained connected with their mentees. Their dedication to these relationships can have a powerful impact on the lives of youth during such a difficult time when human connection has been disrupted. Programming must intentionally include a broader approach to mentoring in order to provide the necessary support for both mentors and mentees, considering needs from how to connect to what online interactions work best for which youth subgroups to providing mentor support. As the world moves through the pandemic without a clear understanding of continued or intermittent closures, e‐mentoring can reach vulnerable youth, offering consistent, knowledgeable, and caring connections. Mentoring should adapt to the needs of not only the mentee but also the changing public health circumstances.

## Conflict of Interest

We have no known conflict of interest to disclose.
